# Where Do They Come From and Where Do They Go? Socioeconomic Patterns in Dog Acquisition and Rehoming

**DOI:** 10.3390/ani14091378

**Published:** 2024-05-03

**Authors:** Tom Kremer, Sue M. Neal

**Affiliations:** 1 Austin Pets Alive, Austin, TX 78703, USA; 2 Department of Political Science, Arkansas State University, Jonesboro, AR 72401, USA; sneal@astate.edu

**Keywords:** animal sheltering, dog acquisition, dog rehoming, income, dogs

## Abstract

**Simple Summary:**

This research looks at how people acquire their dogs, how dogs leave their homes, and how both relate to household income. A survey of 6318 participants in seven distinct communities across the US asked respondents how they acquired their current dogs, how previous dogs left their household, where they left to, and what their income is. We found that, the lower the income, the more likely people obtained their dog through friends and family, while the opposite was true for buying or adopting a dog. At the same time, people in lower-income categories were more likely to give away a dog to friends and family versus to an animal shelter. This means lower-income communities rely more on their friends and family for acquiring and placing their dogs, and we suggest that animal shelters should support these people and their pets to help them stay with those caring for them, rather than solely seeing them as potential adopters.

**Abstract:**

This research examines the ways people acquire dogs in the US as well as the ways the dogs leave the household and the way these differ by income level in seven geographically diverse study communities. A web-based panel survey was distributed and received 6318 responses. Individuals were asked a series of demographic and socioeconomic questions as well as how they acquired their current dogs, how previous dogs left their household, and where they left to. The results indicate that the likelihood of acquiring a dog through friends and family decreased monotonically as income increased, while the opposite was observed for adopting and purchasing a dog. The likelihood of giving a dog away to a friend or family member also decreased as income increased, as opposed to shelter surrender—a person earning over USD 100,000 annually was more than four times likelier to surrender to a shelter than a person earning under USD 15,000. The results suggest a stronger reliance on informal social networks in lower-income communities for both obtaining and placing dogs. As these dogs would otherwise end up in the shelter system, animal shelters may support low-income pet owners to help keep their dogs within their community of care.

## 1. Introduction

Studies citing dog acquisition data in the US mostly refer to the data collected by the American Pet Product Association (APPA) as part of its National Pet Owners Surveys. Data from the 2017–2018 and 2021–2022 editions of the survey are summarized in Table 1 [1,2]. As these data sum to over 100%, they are shown separately from two other sources for dog acquisition data—an American Veterinary Medical Association (AVMA) survey from 2017 to 2018 and a Best Friends Animal Society (BFAS) survey from 2022, shown in the left two columns.

On most accounts, adoption from a shelter or rescue group is the most common source for dog acquisition, while on the AVMA survey, acquisition from a friend, relative, or other individual ranked higher. Breeders and pet stores were next, slightly less common, and finding a stray dog or obtaining one through an existing pet was always under 5%.

One study by Reese and colleagues presented data on differences in ways of acquiring dogs across demographic indicators [5]. As part of a broader survey of Michigan residents conducted in 2017, the participants were asked about the ways they obtained their dogs. Across all respondents, breeder/pet store purchases were more common than adoptions compared to the national data listed above: 35% from breeder/pet stores, 34% from friend/relative/individual, and 20% from shelters/rescues. Additionally, lower-income respondents (less than USD 20,000) were significantly more likely than those in other income groups to have obtained the dog from a family/friend/individual, while respondents with an income of more than USD 150,000 were significantly more likely than those in other income groups to have obtained their dog from a breeder.

In a review of the extant literature around dog acquisition, UK-based authors identified several main categories of factors affecting the decision of where to acquire a dog, including human values and beliefs, desire for a specific type/breed, socioeconomic/demographic background, and challenges with the adoption process [6]. In one study, the decision on where to acquire a dog was found to be related to the person’s feeling of what was the “right thing to do” [7]. In another study focused on preferences around dog acquisition by gender rather than income, women were found to be more likely to acquire an animal from a shelter [8].

Most studies examining dogs and cats leaving the household focus on their relinquishment to shelters, presumably because they are places known to collect information on these animals and their owners, such as the animals’ characteristics and the reasons for their surrender. One study by Weiss et al. (2015) investigated rehoming of animals beyond shelter surrenders in a nationwide phone survey [9]. For dogs, they found the most common destination for rehoming was friends of family (41% of dogs), followed by shelters (34%), strangers (12%), and veterinarians (9%). They also found that the destination was associated with owner age and homeowner status, with both younger people and renters more likely to rehome to friends and less to shelters, but not with annual income (divided only to <USD 50 k and >USD 50 k).

Most of the research did not focus on specific geographic areas, and so variance by geography has not been widely explored. Previous research has also only looked at acquisition alone or disposition alone. Looking only at where people obtain pets in absence of matching information about how pets left the household may leave out important insights.

Previous research has also not situated questions of acquisition or disposition of dogs within a social network context, a potentially key oversight in explaining the differences observed between groups. Social networks refer to the intricate web of social connections and relationships that individuals maintain with others in their social environment. These connections encompass various types of ties, including family, friends, colleagues, and acquaintances, through which individuals exchange information, resources, and support [10]. Social network analysis provides a framework for studying the structure, dynamics, and functions of these connections, focusing on patterns of interaction, communication, and influence within and across social groups [11]. Social networks serve as crucial platforms for socialization, identity formation, and the dissemination of cultural norms and values [12]. Moreover, they play a central role in shaping individual behavior, decision-making processes, and access to opportunities and resources [13]. Overall, social networks constitute the fundamental fabric of social life, influencing various aspects of human interaction and societal organization.

Studies have examined the composition of social networks among low-income individuals, highlighting that these networks often consist of family members, neighbors, and friends from similar socioeconomic backgrounds [14]. These social networks have been found to play a crucial role in resource exchange among low-income populations, including access to job opportunities, financial support, and childcare assistance [15]. Social capital refers to the collective resources embedded within social networks, including trust, reciprocity, and social support, which individuals and groups can leverage for mutual benefit [16,17]. Social capital within low-income communities emphasizes how networks facilitate the exchange of information, trust, and reciprocity, which can have significant implications for individual wellbeing and community resilience [17]. It is, thus, within reason to believe that these same networks potentially provide a similar function in the sourcing and rehoming of companion animals. For example, low-income individuals rely heavily on their social networks for emotional and instrumental support, particularly during times of financial strain or crisis, so it is possible that they seek support from these same networks when struggling to provide for their animals [18].

In order to fill these gaps, this research presents results from a large survey that analyzed both where people obtained their dogs and also how the dogs left the home and to whom they were given.

## 2. Materials and Methods

Seven study communities were chosen based on two sets of criteria—having an animal shelter that participates in the Humane Animal Support Services (HASS) project as a Pilot Organization and being located in different regions in the US. HASS is a national project that is aimed at improving innovation and community collaborations to improve outcomes for animals and people. There are 22 Pilot Organizations (government and non-profit animal shelters) who are active participants in HASS programs. The study communities for the present survey were generally defined by their Metropolitan Statistical Area, except for lower-population areas where they were less likely to achieve the desired sample size. In these places—Cabot, Arkansas, and New Hampshire—larger, general geographic areas were used to achieve the desired sample size. The study communities included Cabot, Arkansas; Dallas, Texas; Fresno, California; Detroit, Michigan; Palm Valley, Texas; and Washington, D.C.

This research used a web panel survey that was electronically administered from June to July of 2021. An external survey company managed the recruitment. The recruitment email did not specify that the survey was about animal ownership, only that it sought to create a representative sample of community members. Upon agreeing to participate, all respondents were asked about the number of pets they currently had and the ways they were acquired. Participants were also asked about the number of pets who left their household and the circumstances of their departure, including the reasons and destinations (see Appendix A for the survey questions). The survey also included several other questions not included as part of this research.

All data analysis was carried out in R (version 4.2.2). A total of 6180 currently owned dogs were reported across 6318 responses. The sources of acquisition were grouped for analysis as follows: “Shelter” and “Rescue group” were grouped into Shelter/Rescue; “Found/Stray” and “Other” responses mentioning “found” or “rescued” were grouped into “Found/Rescued”; “Friend/Family member” and “Other” responses mentioning “Gift”, “Rehome”, and “Neighbor” were grouped into “Friend/Family”; “Bred from our own dog” and “Other” responses mentioning “Litter” or “Born at home” were grouped as “Previous Pet”; “Private breeder”, “Purchased from pet store”, “Craigslist”, and “Other” responses mentioning Facebook or “Purchase” were grouped as “Purchased”. In all, 192 of the 301 “Other” responses were grouped as “Other”. Furthermore, 20 dogs for which the sources were not listed were removed from the analysis. Similarly, 371 dogs from 388 respondents who did not report their income were removed, leaving a total of 5809 dogs summarized in the figures and tables regarding acquisition. Percentages are shown for each value of the examined demographic, e.g., what percentage of people who earn <USD 15 k, USD 15 k–USD 30 k, …, USD 150 k+ acquired their pet through Friend/Family, Shelter/Rescue, and so on for all sources.

In the analysis of the ways pets left homes, the following groups and respective survey wording were used: Ran Away (“Ran away/stolen/lost”), Given Away (as is), Died (“Died of natural causes”), Put to Sleep (“Had to put to sleep”), Other (as is). These data were reported in the survey by person and not by pet—each respondent was able to insert any number of pets who left the household due to each of these reasons, and the percentages shown in the results are out of the total pets reported. People who listed seven or more dogs leaving the household within the last five years were excluded from the analysis (48 of 6318 responses). Moreover, 394 dogs who were from respondents who did not report their income were also excluded. After this filtering, the data contained 4010 dogs who left the home within the previous five years.

In addition, 593 dogs were listed under “Other” as their way of leaving the household, which was a significant percentage of the data. When examining the other reasons provided for them in the free-text field, which were available for 423 of the dogs, many of them were similar enough (and some even identical) to the four existing categories indicated above. The free-text reasons were, thus, manually reviewed by one of the authors and, when appropriate, reclassified into one of the existing categories. Frequently mentioned reasons that were reclassified included dogs dying of “unnatural causes” (e.g., by another animal or person, hit by vehicles—“Died”); moving or housing restrictions (“Given Away”); dogs that were being fostered (removed from the count); and dogs leaving the household with their owner, such as a family member, roommate, or ex-partner (removed from the count). A total of 353 dogs were classified. The final dataset was then summarized by species, race, and income group and reported as percentages.

Data about the destination of pets that were given away were also analyzed. The owners of pets who were reported as being given away were also asked “To whom or where did you give the dog or dogs”. Out of 771 dogs given away left in the data after filtering out respondents who had 7+ dogs leaving the household, 12 animals were missing a destination, leaving 759 dogs in the analysis. The destinations were grouped as follows: “Shelter” and “Rescue group” were grouped as “Shelter/Rescue”, “Friend/Relative” was labeled as “Friend/Family” to match acquisition labels, and “Let loose on farm/wild” was labeled as “Let Loose”. A further 26 responses were examined manually, 9 were classified as Friend/Family, and 17 others were excluded from the analysis for including dogs not actually given away. The remaining dogs were summarized by species and income group and reported as percentages.

In addition to visually showing the percentages of dogs in each income group that were acquired or left the home in a particular way, the odds ratios were calculated within each category relative to the lowest-income group of <15 k. For example, for acquisition, Table 2 shows, for each way of acquiring a dog, the odds ratios for being acquired this way for people of each income group over <15 k (which is the baseline, OR = 1).

## 3. Results

### 3.1. Acquisition

**Figure 1:**
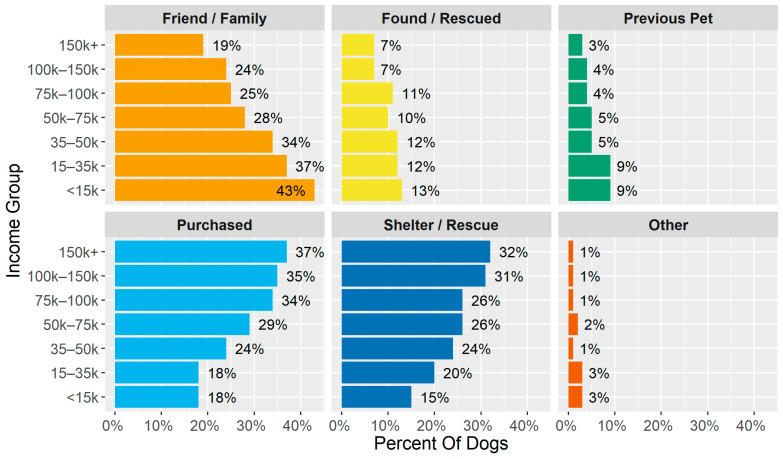
Percentage of dogs acquired in different ways by income group (for example 19% of people who earn over USD 150,000 acquired their dog from a friend or family member).

Figure 1 shows what percentage of each income group acquired dogs in the various methods. On the aggregate, acquiring dogs from Friend/Family decreased monotonically as income increased, from 43% of dogs for the lowest-income group to 19% for highest. Conversely, purchasing (18% for the lowest to 37% for the highest-income group) and adopting (15% to 32%) increased with income.

As another way of examining the differences in the ways of acquisition between the income groups, Table 2 shows the odds ratio for acquiring a dog in a given method (columns) for each income group, relative to the baseline of <USD 15,000.

**Table 2:** Odds ratio for acquiring a dog from a given source by income group.

Income Group	Friend/Family	Found/Rescued	Previous Pet	Purchased	Shelter/Rescue	Income Group
<15 k (baseline)	1.00	1.00	1.00	1.00	1.00	<15 k (baseline)
15–35 k	0.80	0.96	0.97	1.05	1.45	15–35 k
35–50 k	0.67	0.87	0.54	1.42	1.83	35–50 k
50 k–75 k	0.50	0.75	0.53	1.89	2.04	50 k–75 k
75 k–100 k	0.42	0.79	0.40	2.34	2.02	75 k–100 k
100 k–150 k	0.40	0.46	0.35	2.43	2.53	100 k–150 k
150 k+	0.30	0.52	0.31	2.73	2.74	150 k+

**Figure 2:**
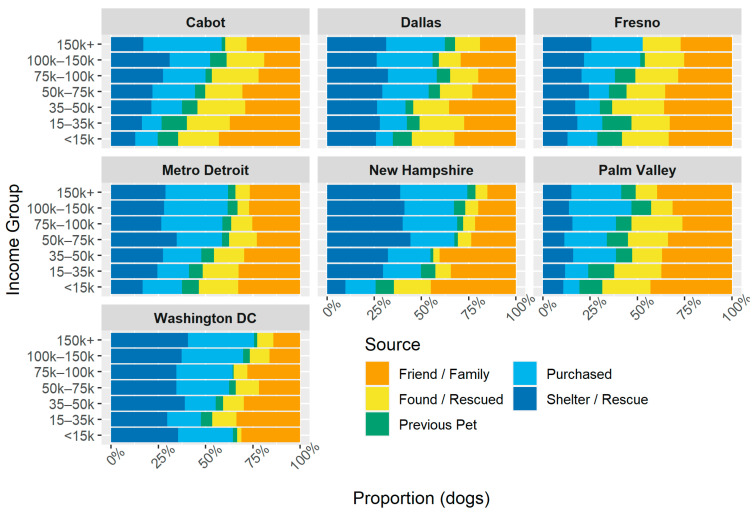
Distribution of ways of acquisition within each study community and income group.

This pattern was also observed when examining the ways of acquiring pets by income group within each study community, with minor variations, as shown in Figure 2. In all communities, Friend/Family as a source decreased in frequency as income increased, while the combined share of Purchased and Shelter/Rescue increased. In most places, the increase in purchasing was more pronounced than that in adopting. Found/Rescued and Previous Pet were relatively stable across income groups. “Other” was removed from this visualization for simplicity as it was consistently less than 3%.

### 3.2. Disposition

**Figure 3:**
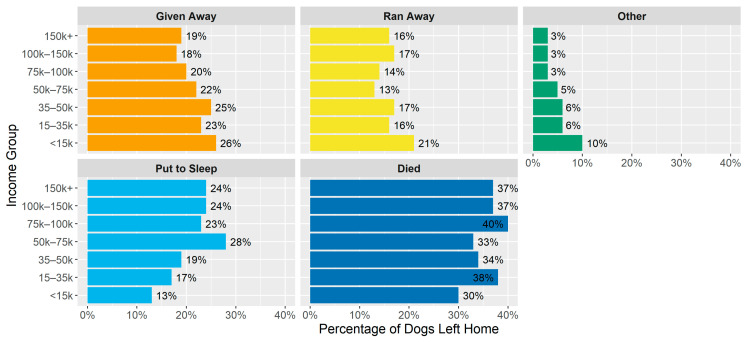
Distribution of ways dogs left the household by income group.

Unlike acquisition, the ways of dogs leaving the home were not as different between income groups, as shown in Figure 3 and Table 3 (odds ratios). There was a small decrease in the percentages of pets running away and being given away and an increase in animals dying and being put to sleep as income increased, but they were fairly minor.

**Figure 4:**
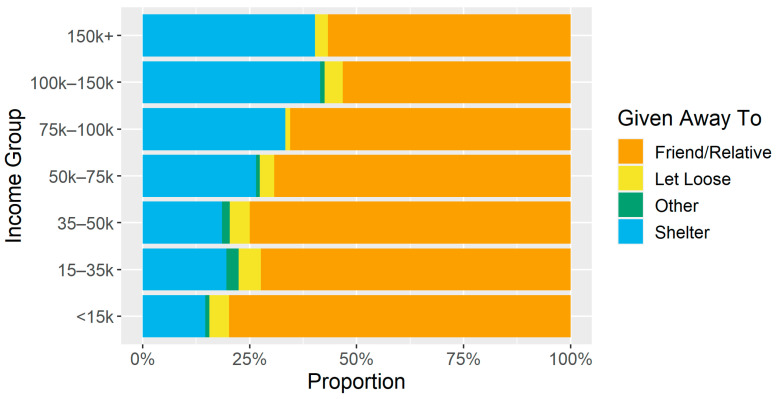
Distribution of places dogs were given away to by income group.

When looking at the destinations of dogs given away, the higher-income groups had fewer dogs going to friends or relatives, while the opposite was true for the lower-income tiers (Figure 4, Table 4). In the <15 k income group, 80% of dogs given away went to friends or relatives while 15% were surrendered to a shelter; for those earning 150 k+, 57% of dogs were given away to friends/relatives while 40% were surrendered. These data are not shown within each study community because when dividing by both income groups and study communities, several of these subgroups contain a single-digit number of dogs.

## 4. Discussion

Our results provide new insights into the pathways through which dogs are obtained by and leave the households of individuals in the seven study communities. The most notable findings indicate that lower-income individuals are more likely to obtain their animal through friends and family and less likely to purchase or adopt a dog. Similarly, lower-income respondents were more likely to give a dog away to friends or family as opposed to surrendering them to an animal shelter. This may be reflective of resource constraints and/or the reliance of lower-income individuals on informal social networks as opposed to formal government systems for obtaining or relinquishing a dog.

Comparing these results to the previous surveys presented above, the AVMA results are fairly consistent with the results of this survey, with percentages falling in the middle of the income tiers for adoptions and purchases, while acquisition through friend/family was more frequent than in this survey and finding/rescuing less frequent. The BFAS survey results, which were different from those of the AVMA, are also different from these results, as they reported a much higher adoption frequency (39%, higher than any income category reported here). Comparing these results to the APPA surveys is more challenging because questions about the ways of acquisition in these surveys were multiple response and summed to over 100%. The fact that both the BFAS and AVMA surveys were carried out nationally while our survey more deeply explored a handful of individual communities may explain some of the differences observed.

Unlike Weiss and colleagues who found no relationship between the destination of dogs given away and income, the data from this survey showed that higher-income respondents were less likely to rehome their dogs to friends or family and more likely to rehome them to shelters than lower-income respondents (and vice versa). One difference between the surveys is the more granular income grouping in this study, but since the percentage of friend/family rehoming decreased gradually with income, a difference would show even if participants were only divided into <50 k and >50 k groups. The overall percentage of rehoming to shelters was a bit lower than that in the previous study, while that of friends/relatives was higher, but the lack of clear “vet” and “stranger” groups, which together accounted for 21% of rehomings, makes the comparison imperfect. That survey relied on a methodology of random digit dialing, which may reach different participants than web panels used in our research.

The more notable patterns corresponding to income groups appear in the ways the dogs were acquired as well as in where they were given away to, while smaller differences appeared in the ways they left the household. For the latter, people in lower-income categories had their animals run away or had to give up their animals more and had their pets leave the household due to death (natural, unnatural, or euthanasia) less often than higher-income people. Having animals leave the home by running away may be a consequence of an inability to afford appropriate enclosures such as fencing. Further, having animals leave the home prior to end of life may mostly be a function of the instability in the lives of lower-income individuals. Lastly, variation in the options at end of life, such as choosing euthanasia, may also be resource constrained due to the cost and accessibility challenges related to euthanasia.

A key finding of this research is that there are similar patterns related to where lower-income communities acquire dogs and where they choose to place them if they need to rehome them. To quantify the differences, lower-income individuals acquired pets from others in their community more frequently than higher-income individuals, with the highest-income groups being about three times more likely to acquire a dog from a shelter and three times less likely to acquire a dog from a friend or relative compared the lowest-income groups. Lower-income owners also rehomed dogs to friends and family as opposed to a shelter more often than higher-income individuals—the highest-income groups were four times more likely to rehome a dog to a shelter and four times less likely to rehome it to a friend or relative compared to the lowest-income groups.

We may conceive of this finding as reflecting a social network functioning for both obtaining and placing companion dogs in lower-income communities. Social capital is the reciprocity and collective action (among other attributes) that are present among individuals in a community [16]. Research has found that lower-income individuals build this type of social capital out of necessity as a coping mechanism for the instability and daily challenges of living within substantial resource constraints [19]. By operating outside of the traditional sheltering environment, the individuals in our study communities may have been exhibiting this sort of self-economy in which the community is both the source of a companion animal as well as the destination when one is no longer wanting or able to keep a dog. This reliance on informal social networks, as opposed to government/nonprofit administered animal shelters is an important insight for shelters working to interface with and support all members of their community.

Another way to frame this finding is that animal shelters, meant to serve as a safety need for pets without a caregiver, end up serving more often people who otherwise have more resources (judging based on income) both for acquiring and giving up pets, while people from lower-income groups rely more frequently on their communities. This interpretation is consistent with social network theory and its particularly important role in lower-income communities. Reliance on the informal social network is likely not the only factor impacting how income predicts interaction with formal resources, such as shelters. For example, the observation that higher-income people tended to adopt more might also make them more aware of the shelter’s resources and services when in need of rehoming a dog.

Animal shelters may act on the data showing the lower percentages of people in lower-income categories adopting dogs from shelters by trying to increase adoption rates among people in lower-income groups by reducing adoption fees and promoting programs in that spirit. While they could benefit from these programs, shelters may also recognize that dogs given to and received from friends and family members are ones that without the receivers’ support would end up at the shelter system; they may further recognize that there are benefits to the pet staying closer to its original home, and that instead of seeing them only as potential adopters, shelters may choose to focus on supporting these pet owners with programs that would help keep these pets with their people. This support may include services that would normally be associated with adoption such as healthcare, behavioral training, vaccinations, and sterilization. If shelters were still interested in increasing their adoption pool, rather than thinking about people who might otherwise obtain a pet from somebody they know, they may target those who might otherwise buy a pet from a store or a breeder, which are generally not animals that would otherwise reach the shelter system. This has various implications for the kinds of programs shelters may carry out to increase adoptions, understanding what brings people to buy rather than adopt animals, as opposed to strictly lowering fees. Of course, this does not mean that, in some communities, people in lower-income categories do not avoid adopting because of high fees or similar reasons related to the adoption process, and such measures may still have their place as well.

This research has a few notable limitations. First, the survey was focused on specific geographic areas where a shelter participating in the HASS project was present. Thus, these results may not be generalizable to the entire country. There are also potential biases inherent in the survey methodology used. Relying on web panels may miss certain segments of the population such as those without access to the internet. The survey was conducted in English only, which leaves out populations who do not read English well or at all. Future research should continue to explore the cycling of animals in a community outside of shelters and how shelters could best support these activities. This could be expanded to include more qualitative research to understand the lived experiences of individuals that may inform where they choose to acquire an animal or where they choose to rehome an animal.

## 5. Conclusions

Differences between survey populations, survey methodologies, and the point in time at which a survey is administered provide inconsistent conclusions regarding the impact of factors like income on the acquisition sources for dogs. This research has added to the understanding of dog acquisition and provides additional insight by looking at both the acquisition and where animals were given away to. The story that emerges from the analysis hints at activity among lower-income residents that largely takes place outside of the traditional sheltering environment. Dogs were more likely to be acquired from social connections and were more likely to be similarly given away through social connections. This information could help inform shelter operations where, instead of targeting these community members with low cost or free animals, they may consider other ways to support this existing network of animal exchange. Ensuring that these individuals have access to support services such as vaccination and spay/neuter were identified as key ways in which shelters could accomplish that goal. This would allow the community to continue to function as their own support systems for rehoming and acquiring animals outside of the shelter environment, which is likely better for the behavioral and medical welfare of the animals.

## Figures and Tables

**Table 1:** Summary of where dogs are acquired from.

Source	AVMA 2017–2018 [3]	BFAS 2022 [4]	APPA 2017–2018 * [1]	APPA 2021–2022 * [2]
Adopted—shelter/rescue/pet store	28%	39%	44%	40%
Friend/relative/another individual	37%	27%	35%	31%
Breeder/pet store	28%	23%	29%	30%
Found/stray	5%	5%	4%	4%
Previous pet	2%	NA	1%	4%

* Numbers sum to more than 100% because multiple responses were accepted.

**Table 3:** Odds ratio for ways of dogs leaving the household by income group.

Income Group	Given Away	Ran Away	Put to Sleep	Died	Other
<15 k (baseline)	1.00	1.00	1.00	1.00	1.00
15–35 k	0.86	0.72	1.40	1.40	0.56
35–50 k	0.93	0.75	1.56	1.18	0.60
50 k–75 k	0.79	0.56	2.61	1.12	0.45
75 k–100 k	0.68	0.62	2.04	1.54	0.31
100 k–150 k	0.62	0.80	2.21	1.35	0.27
150 k+	0.68	0.74	2.20	1.35	0.25

**Table 4:** Odds ratio for dogs given away to friend/relative and shelter by income group.

Income Group	Friend/Relative	Shelter
<15 k (baseline)	1.00	1.00
15–35 k	0.68	1.47
35–50 k	0.74	1.34
50 k–75 k	0.47	2.13
75 k–100 k	0.36	2.81
100 k–150 k	0.24	4.24
150 k+	0.23	4.32

## Data Availability

The raw data supporting the conclusions of this article will be made available by the authors on request.

## Appendix A. Survey Questions Used

- How many dogs, if any, do you currently have in your household? [0, 1, 2, …, 6+]
- How did [each of up to three dogs mentioned] join your household?
  ○ Found/stray
  ○ Friend/family member
  ○ Shelter
  ○ Rescue group
  ○ Bred from our own [dog/cat]
  ○ Private breeder
  ○ Purchased from pet store
  ○ Craigslist
  ○ Other (please specify)
- How many dogs, if any, have left the household within the past 5 years?
  # of dogs left household in past 5 years: _________ [0–99]
- [If dogs > 0] thinking about the dog or dogs that left the household within the past 5 years, how many dogs are no longer in the household because…
  ○ They ran away/stolen/lost [0–99]
  ○ They were given away [0–99]
  ○ Died of natural causes [0–99]
  ○ Had to put to sleep [0–99]
  ○ Some other reason [0–99]
    ■ [If > 0] What were the other reason(s) the dog or dogs are no longer in the household? [free text]
- [If “they were given away” > 0] To whom or where did you give the dog or dogs? Please check all that apply. [checkbox]

	Dog 1	Dog 2	Dog 3	Dog 4
Friend/relative	[ ]	[ ]	[ ]	[ ]
Rescue group	[ ]	[ ]	[ ]	[ ]
Shelter	[ ]	[ ]	[ ]	[ ]
Let loose on farm/wild	[ ]	[ ]	[ ]	[ ]
Other (specify) [free text]	[ ]	[ ]	[ ]	[ ]

## References

[1] American Pet Products Association (2019). The 2017–2018 APPA National Pet Owners Survey.

[2] American Pet Products Association (2022). The 2021–2022 APPA National Pet Owners Survey.

[3] American Veterinary Medical Association AVMA (2018). Pet Ownership and Demographics Sourcebook.

[4] (2022). Best Friends Consumer Adoption Research Analysis. https://network.bestfriends.org/sites/default/files/inline-files/ConsumerAdoptionAnalysis_full_version.7.14.2022.pdf.

[5] Reese L.A., Skidmore M., Dyar W., Rosebrook E. (2017). No Dog Left Behind: A Hedonic Pricing Model for Animal Shelters. J. Appl. Anim. Welf. Sci..

[6] Holland K.E. (2019). Acquiring a Pet Dog: A Review of Factors Affecting the Decision-Making of Prospective Dog Owners. Animals.

[7] Bir C., Widmar N., Croney C. (2016). Public Perceptions of Dog Acquisition: Sources, Rationales and Expenditures.

[8] Bir C., Widmar N.J.O., Croney C.C. (2017). Stated Preferences for Dog Characteristics and Sources of Acquisition. Animals.

[9] Weiss E., Gramann S., Spain C.V., Slater M. (2015). Goodbye to a Good Friend: An Exploration of the Re-Homing of Cats and Dogs in the U.S. Open J. Anim. Sci..

[10] Scott J., McLevey J., Carrington P.J. (2011). The Sage Handbook of Social Network Analysis.

[11] Wasserman S., Faust K. (2012). Social Network Analysis.

[12] Granovetter M.S. (1973). The Strength of Weak Ties. Am. J. Sociol..

[13] Burt R. (2007). Brokerage & Closure: An Introduction to Social Capital.

[14] Lin N. (2011). Social Networks and Status Attainment. The Sage Handbook of Social Network Analysis.

[15] Edin K., Kefalas M. (2011). Promises I Can Keep: Why Poor Women Put Motherhood before Marriage.

[16] Bhandari H., Yasunobu K. (2009). What Is Social Capital? A Comprehensive Review of the Concept. Asian J. Soc. Sci..

[17] Portes A. (1998). Social Capital: Its Origins and Applications in Modern Sociology. Annu. Rev. Sociol..

[18] Stack C.B. (1997). All Our Kin: Strategies for Survival in a Black Community.

[19] Piff P.K., Kraus M.W., Côté S., Cheng B.H., Keltner D. (2010). Having Less, Giving More: The Influence of Social Class on Prosocial Behavior. J. Personal. Soc. Psychol..

